# Evaluation of salivary electrolytes during estrous cycle in Murrah buffaloes with reference to estrus detection

**DOI:** 10.14202/vetworld.2016.1157-1161

**Published:** 2016-10-28

**Authors:** Indu Devi, Pawan Singh, Surerder Singh Lathwal, A. Kumaresan, Kuldeep Dudi

**Affiliations:** 1 Livestock Production Management Section, ICAR - National Dairy Research Institute, Karnal - 132 001, Haryana, India; 2 Department of Animal Reproduction (Livestock Production Management), Livestock Research Centre, ICAR - National Dairy Research Institute, Karnal - 132 001, Haryana, India; 3 Animal Nutrition Group, National Dairy Development Board, SCF-80, Panchkula - 134 109, Haryana, India

**Keywords:** heat detection, noninvasive method, saliva electrolytes, silent heat

## Abstract

**Aim:**

Timely estrus detection is one of the critical factors for increasing reproductive efficiency in buffaloes. In recent decades, saliva has become a more popular as a noninvasive source for determining physiological status of animals by various biochemical electrolytes. This study was designed to assess and correlate changes in different salivary minerals concentration (calcium, inorganic phosphorus, magnesium, sodium, potassium, and chloride) during different stages of the estrous cycle in Murrah buffaloes.

**Materials and Methods:**

The saliva samples were collected during the different phases of the estrous cycle from 20 Murrah buffaloes in early morning hours and were assayed using respective minerals assay kits.

**Results:**

The concentrations of calcium (8.76±0.08-12.11±0.11 mg/dl), inorganic phosphorus (6.56±0.13-14.72±4.50 mg/dl), magnesium (2.27±0.14-5.79±0.15 mg/dl), sodium (139.47±0.31-159.62±1.22 mmol/L), potassium (12.40±0.22-26.85±1.22 mmol/L), and chloride (109.28±0.41-137.07±0.68 mmol/L) varied during the different phases of estrous cycle. The concentration of calcium, inorganic phosphorus, magnesium, sodium, potassium, and chloride in saliva were significantly (p<0.01) higher during estrus phase compared to other phases of the estrous cycle. All these minerals were positively and significantly (p<0.0001) related to estrogen concentration while salivary concentrations of calcium, magnesium, sodium, and chloride showed a significant (p<0.0001) negative correlation with progesterone level in blood plasma.

**Conclusion:**

These preliminary findings indicate that there are definite variations in salivary mineral and electrolyte concentrations during different phases of the estrous cycle. These results may be used as an aid for estrus detection/confirmation in buffaloes although validation of the results using a large number of animals is required.

## Introduction

Buffaloes have played a prime role in milk production, meat industries, and other agricultural activities like draught production throughout the known Indian history. At present, Indian buffaloes have vital influence in dairy industry development and contribute about 52% share in total milk production of India [1], 68% of worldwide buffalo milk production [2] along with 42.45% contribution to global buffalo meat, making India the largest milk producer and exporter of carabeef in world [3]. The milch buffaloes increased from 48.64 to 51.05 million with an increase of 4.95% from 2007 to 2012 census [4]. In spite of having all potential of becoming best dairy and meat industry animal, estrus detection in this species is difficult due to high expression of silent heat, poor expression of estrus behavior [5]. Owing to these factors low reproductive efficiency in buffaloes remains a major economic problem. To overcome this problem, there is an increasing demand for noninvasive test to predict the fertile period during estrous cycle.

Cyclic changes in various physical properties and biochemical constituents of body fluids may reflect the hormonal changes associated with estrous cycle and may be utilized clinically to determine the stage of estrous cycle. A variety of salivary electrolytes such as sodium, potassium, magnesium, calcium, and inorganic phosphorus have been found to fluctuate during the periovulatory period of cycle. The cyclic physiological changes are mainly brought about by the ovarian hormones; estrogen and progesterone, the levels of which show variation during the estrous cycle. Effect and correlation of ovarian hormones on water and electrolyte balance has been well documented [6]. Estrogen leads to a marked acceleration of calcium uptake and decrease of its elimination through gut [7]. Theoretically, the ferning (crystallization) pattern of saliva coincides with the female fertile period in women. The ferning is caused by NaCl, which cyclically increases under the influence of estrogen [8]. Progesterone reportedly has a natriuretic effect [9], and the increase in progesterone after ovulation is thought to be followed by a compensatory rise in aldosterone concentration and subsequent effects on levels of electrolytes such as sodium, potassium, and calcium. Magnesium has also been found to have its role in regulation of menstrual function, along with its role in basal metabolism that changes over the course of the menstrual cycle in women [10].

With this backdrop, this study has been envisaged to evaluate the proportional changes in salivary minerals such as calcium, magnesium, sodium, potassium, and inorganic phosphorus during different phases of estrous cycle in Murrah buffaloes.

## Materials and Methods

### Ethical approval

This study was duly approved by the Institutional Animal Ethics Committee, ICAR - National Dairy Research Institute, Karnal, Haryana, India.

### Experimental animals

A total of 20 normally cyclic and healthy Murrah buffaloes (*Bubalus bubalis*) free from any anatomical disorder and/or reproductive disabilities and diseases maintained at the Livestock Research Centre, NDRI, Karnal, Haryana, India, were selected as an experimental animal in this investigation. These buffaloes were kept separately in a loose housing system within a premise of 240 m^2^ covered and open area (as per BIS). The main purpose of the above said housing system was to provide enough space for their free movement and better observation of signs of estrus. The animals were fed *ad libitum* with conventional diet as per the normal feeding schedule being followed for buffaloes.

### Confirmation of estrus and sample collection

The buffaloes were carefully monitored for estrus signs including vulva swelling and reddening, vaginal mucous discharge, and restlessness. In addition, the exhibition of male behavior toward estrus buffalo such as Flehmen reaction, vaginal licking and mounting was taken into account to confirm the estrus phase. Finally, the estrus was confirmed by rectal palpation by experienced veterinarian. Saliva and blood samples were collected continuously for 25 days between 6.00 and 8.00 a.m. (before feeding).

Once 2^nd^ estrus was confirmed, the collected samples were categorized as proestrus (−3 to −1 days), estrus (0 day), metestrus (1-2 days), and diestrus (14-21 days) phases. Collected saliva samples were centrifuged at 3000 rpm for 15 min at 4°C to remove any feed particle, etc. The separated saliva samples were stored in cryovials at −20°C. The electrolytes were estimated at the Central Institute for Research on Buffaloes, Hisar (Haryana) using mineral specific assay kits. The blood sample was centrifuged at 4°C at the rate of 3000 rpm for 20 min to separate the plasma and stored at −20°C.

### Electrolytes and hormones estimation

Calcium estimation was done by Calcium Kit (OCPC Method). Phosphorus estimation was done by Phosphorus kit (Molybdate U.V method). Sodium estimation was done by colorimetric method (Modified Maruna and Trinder’s method). Potassium estimation was done by colorimetric method based on Turbidimetric method. Magnesium estimation was done by Magnesium kit (Calmagite method) and chloride estimation was done by Chloride kit (Thiocyanate method). The hormones were estimated using E2 and P4 bovine ELISA test kits (Endocrine Technologies, Inc., Newark, CA).

### Statistical analysis

The data thus generated were subjected to Statistical Analysis Systemsoftware package to find out the significant difference in salivary electrolytes and plasma endocrine profile of Murrah buffaloes under different stages of estrous cycle.

## Results and Discussion

**Figure-1:**
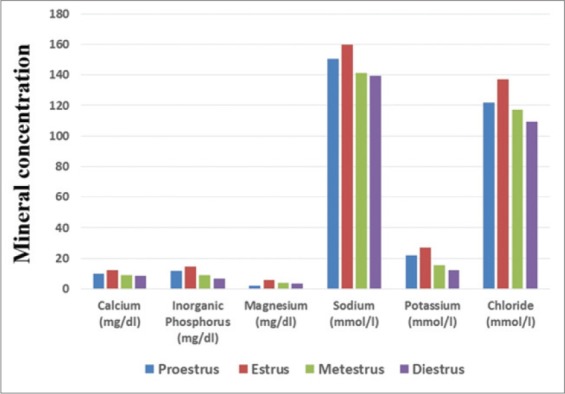
The salivary electrolytes concentration during estrous cycle in Murrah buffaloes.

The levels of saliva electrolytes such as calcium (12.11±0.11 mg/dl), inorganic phosphorus (14.72±4.5 mg/dl), magnesium (5.79±0.15 mg/dl), sodium (159.62±1.22 mmol/L), potassium (26.85±1.22 mmol/L), and chloride (137.07±0.68 mmol/L) were observed to be significantly (p<0.01) higher during estrus phase compared to all other phases of estrous cycle (Table-1 and Figure-1). Inorganic phosphorus and calcium gradually decreased from estrus to diestrus phase. Magnesium was found highest in estrus phase, but lowest in proestrus phase. During the estrus period, sodium, potassium and chloride increased significantly (p<0.01) as compared to metestrus and diestrus phases of the estrous cycle.

**Table-1:** Mean±SE values of salivary electrolytes during different phases of estrous cycle in Murrah buffaloes.

Parameters	Phases of estrous cycle			

	Proestrus	Estrus	Metestrus	Diestrus
Calcium (mg/dl)	9.95±0.11^C^	12.11±0.11^A^	8.81±0.08^B^	8.76±0.08^C^
Inorganic phosphorus (mg/dl)	11.80±0.21^B^	14.72±4.5^A^	9.08±0.0.17^B^	6.56±0.13^B^
Magnesium (mg/dl)	2.27±0.14^B^	5.79±0.15^A^	3.92±0.12^C^	3.28±0.5^D^
Sodium (mmol/L)	150.30±0.32^B^	159.62±1.22^A^	141.40±0.90^C^	139.47±0.31^D^
Potassium (mmol/L)	22.16±0.33^B^	26.85±1.22^A^	15.29±0.34^C^	12.40±0.22^D^
Chloride (mmol/L)	121.98±0.77^B^	137.07±0.68^A^	117.12±1.08^C^	109.28±0.41^D^

Values bearing different superscripts in a row differ significantly (p<0.01). SE=Standard error

The least square means of plasma estradiol and progesterone levels during proestrus, estrus, metestrus, and diestrus phases of estrous cycle were observed to be 29.46±0.65, 19.35±0.35, 26.68±0.19, and 5.43±0.13 pg/ml, respectively, and 1.01±0.90, 0.57±0.20, 1.25±0.30, and 2.59±0.12 ng/ml, respectively (Table-2). The level of blood progesterone hormone was significantly (p<0.01) lower in estrus phase as compared to other phases. A significant correlation was found between saliva electrolytes and estradiol (Table-3).

**Table-2:** Mean±SE values for blood plasma hormones levels during different phases of estrous cycle in Murrah buffaloes.

Hormones	Phases of estrous cycle			

	Proestrus	Estrus	Metestrus	Diestrus
Estrogen (pg/ml)	29.46±0.65^A^	19.35±0.35^B^	6.67±0.19^C^	5.43±0.13^D^
Progesterone (ng/ml)	1.01±0.90^B^	0.56±0.20^C^	1.24±0.30^B^	2.58±0.12^A^

Values bearing different superscripts in a row differ significantly (p<0.01). SE=Standard error

**Table-3:** Pearson’s correlation coefficients of saliva electrolytes and blood plasma hormones.

Hormones	Different electrolytes					

	Calcium	Phosphorus	Magnesium	Sodium	Potassium	Chloride
Estrogen	0.67^A^	0.63^A^	0.62^A^	0.49^A^	0.59^A^	0.70^A^
Progesterone	−0.50^A^	−0.23	−0.46^A^	−0.37^A^	−0.23	−0.57^A^

Values bearing superscript “A” are significantly correlated (p<0.0001)

The cyclic hormonal changes can affect a variety of physiological and biochemical processes. There are very few reports on the changes in salivary calcium, inorganic phosphorus, magnesium, sodium, potassium, and chloride levels in various phases of the estrous cycle in buffaloes. This investigation revealed that salivary electrolytes considerably varied depending on the reproductive status of animals. Peak serum calcium concentration observed at day 0 (estrus phase) may be due in part to high serum concentration of estradiol recorded during this phase. Similar observations were reported [11] in mares during the estrus phase of the estrous cycle. In addition, the ability of estradiol to retain salt and to alter ion transport in various other epithelial cells could be partly responsible for high saliva calcium observed during the estrus phase. It is reported that estrogen causes increase in parathyroid activity [12] which leads to marked acceleration of calcium uptake [7] and decreases of its elimination from pigeon’s gut [13]. This increase in serum calcium level in estrus phase may be necessary to support the increased neuromuscular activity, and ovarian hormone synthesis and release associated with this phase of estrous cycle. Changes in progesterone concentrations are not correlated with the changes in salivary electrolytes during cycle, suggesting that this hormone does not account for the change between the preovulatory phase and the postovulatory phase.

In a similar research carried in dogs, level of salivary magnesium (Mg) has been reported to be highest during estrus and least during proestrus phase [14]. The significant(p<0.01) increase in salivary sodium, potassium, and chloride levels was seen during proestrus and estrus phases compared to postovulatory phases (diestrus phase). Possible cause for this change in sodium concentration includes the increased concentrations of antidiuretic hormone in the postovulatory phase [15], or of other steroid hormones. The possible reason for increased levels of these minerals might be their positive correlation with estrogen hormone as compared to progesterone. The fact that the change in plasma sodium is not associated with changes in weight or in concentration of urea, creatinine or albumin suggests that total body water and intravascular volume remain constant. Thus, it appears that sodium is lost in excess of water in the period of ovulation. It is known that saliva ferning depends principally on the electrolytes concentration (especially NaCl, KCl, CaCl_2_) and chemo-physical properties of the mucins it contains (sialic acid) [16,17]. The estrogens increase the water content in mid-cycle and determine the most favorable condition, optimal proportion of water and optimal amounts of salts and sialomucin [18].

The level of estrogen hormone in blood was significantly (p<0.01) higher during proestrus phase than other phases of the estrous cycle in Murrah buffaloes. These hormonal findings were found in agreement with previous studies [19]. Peripheral P_4_ concentrations are lower on the day of estrus, rise to peak concentrations of 1.6-3.6 ng/ml on days 13-15 of cycle [20] or even more on day 17^th^ [21] before declining to basal levels at the onset of next estrous cycle. The similar pattern was observed in the present investigation.

Finally, along with the prevailing known factors affecting estrus expression and mechanisms for heat detection [22,23] new initiatives need to be taken and further research in this area should focus on identifying the biochemical mechanisms triggering changes in electrolyte metabolism and in mucin expression in saliva, all of which could explain the observed variations in electrolytes patterns. It is also of importance to elucidate the mechanism through which changes in sex steroid levels influence the different electrolytes observed among healthy buffaloes and in those suffering from reproductive disorders.

## Conclusion

This study showed possibility of quantifying the salivary electrolytes such as sodium, potassium, calcium, magnesium, and inorganic phosphorus levels in saliva. As the increased activity of minerals tended to coincide significantly with the increase in plasma hormones like estradiol, it may be possible to use saliva samples for differentiation of stages of estrous cycle. Further, this study may open new ways to provide a non-invasive biological fluid to understand the relation of widely prevailing mineral deficiency in dairy animals and the level of various reproductive hormones in body.

## Authors’ Contributions

Research work was done by ID. The experiment was designed and supervised by PS. SSL assisted ID in all technical support and data recording and KD assisted in literature collection and data analysis. PS and SSL provided valuable suggestion regarding design of experiment and data analysis. ID and KD compiled the results. KD and AK assisted in manuscript preparation. All authors have read and approved the final manuscript.
